# Oral myopericytoma: a rare pediatric case report and a review of the literature

**DOI:** 10.1186/s12903-021-01534-y

**Published:** 2021-04-07

**Authors:** Dalit Porat Ben Amy, Victoria Yaffe, Rawan Kawar, Sharon Akrish, Imad Abu El-Naaj

**Affiliations:** 1 Oral Medicine Unit, Baruch Padeh Medical Center, 15208 Poriya, Lower Galilee, Israel; 2 The Azrieli Faculty of Medicine in the Galilee, Bar Ilan University, 1311502 Safed, Israel; 3 Department of Oral and Maxillofacial Surgery, Baruch Padeh Medical Center, 15208 Poriya, Lower Galilee, Israel; 4 Department of Periodontology and Oral Implantology, School of Dental Medicine, Tel Aviv University, Tel-Aviv, Israel; 5 Department of Oral and Maxillofacial Surgery, Rambam Medical Center, Haifa, Israel; 6 Department of Pathology, Rambam Medical Center, Haifa, Israel; 7 Technion School of Medicine, Haifa, Israel; 8 The Azrieli Faculty of Medicine in the Galilee, Bar Ilan University, 1311502 Safed, Israel

**Keywords:** Oral myopericytoma, Conservative treatment, Pediatric pathology, Case report

## Abstract

**Background:**

Myopericytoma is a rare mesenchymal neoplasm with perivascular myoid differentiation that arises most commonly in middle adulthood. The lesion generally involves the subcutaneous tissue of distal extremities. Myopericytoma of the oral cavity is extremely rare. Herein we report a case of oral myopericytoma in a pediatric patient, who was treated via a conservative approach with a follow up of 8 years. The case is followed by a literature review. To our knowledge this is the first documented case of oral myopericytoma affecting a patient of such a young age.

**Case presentation:**

A 6 years old boy was referred to the maxillofacial surgery department for the evaluation of a solitary growth of the right maxillary buccal and palatal gingiva. Histology and immunohistochemistry confirmed the diagnosis of myopericytoma.

**Conclusions:**

Our patient was treated by local excision with no recurrence in 8 years of follow up. Conservative approach should be considered for the treatment oral myopericytoma especially in young patients in tooth bearing areas.

## Background

Myopericytoma (MPC) was first described in 1996 as a slow-growing, well-circumscribed nodule of 2 cm or less, affecting mostly the skin and superficial soft tissues of distal extremities in adults. Recently, few rare cases of head and neck MPCs were described [1–3].

MPC can be diagnosed by histology of the biopsied tissue. However, owing to overlapping morphologic features, MPC must be distinguished from other solitary fibrous tumors [4] using immunohistochemistry, i.e., positive staining for smooth muscle actin, smooth muscle myosin heavy chain, h-caldesmon and calponin [3, 5–8] and negative for desmin [9].

Most cases of MPC are benign. Nonetheless, few malignant and /or recurring cases were described [10, 11]. In the oral cavity only one case of malignant MPC was reported [2].

A detailed case report of a 6 year old patient diagnosed with oral MPC is described, followed by a literature review.

## Case presentation

A 6 years old boy, ASA 1 (American Society of Anesthesiologists physical status 1) was referred to a maxillofacial surgeon in November 2012, for the evaluation of a solitary growth of the posterior right maxilla.

Oral examination revealed a reddish, well-circumscribed and firm nodule that measured in its largest diameter about 2 cm. The mother of the patient noticed the lesion about a month before the initial examination. Main complaint upon presentation was bleeding from the area of the lesion while brushing the teeth.

**Fig. 1:**
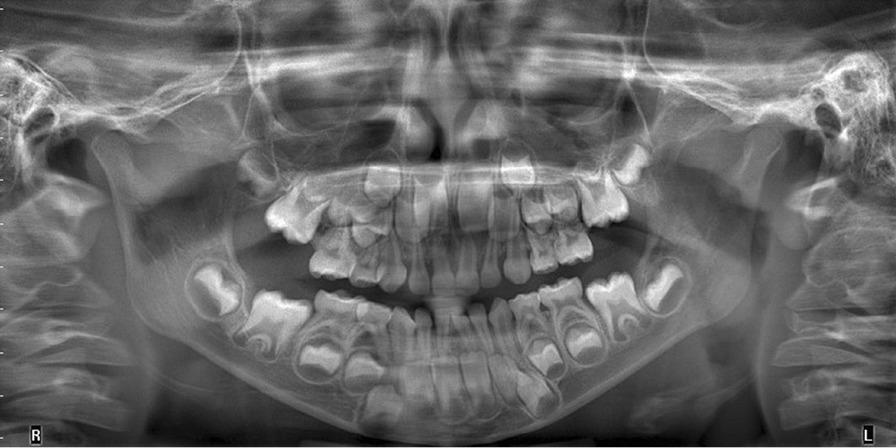
Panoramic X-ray at initial examination failed to demonstrate the lesion or any bone involvement/destruction

In order to establish better differential diagnosis and to undercover whether the lesion originates in the bone (meaning a central epicenter), causing bone destruction or whether it is a lesion of the soft tissue alone, the patient was sent to perform imaging. A Panoramic X-ray (Fig. 1) and cone beam computed tomography (CBCT) failed to show any pathological change of the right maxilla.

**Fig. 2:**
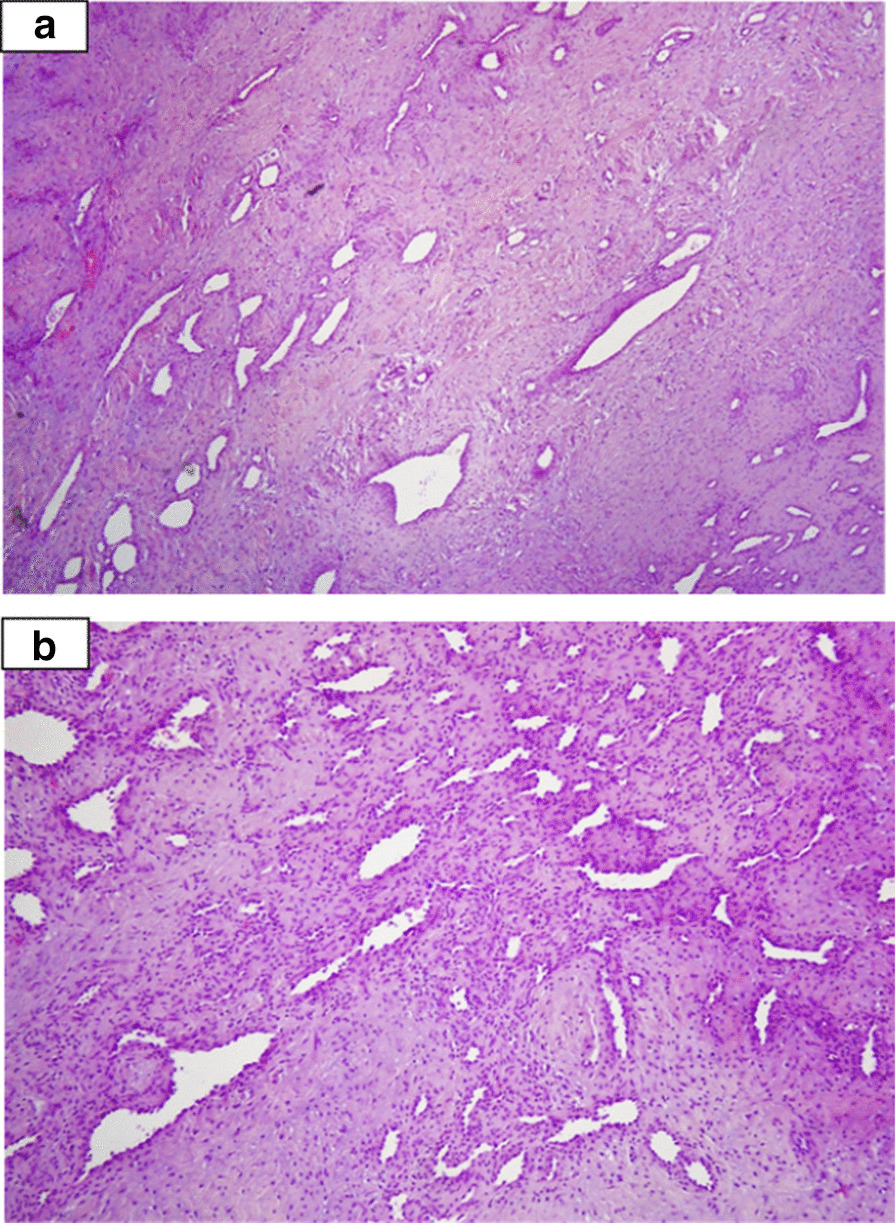
(**a**) Proliferating nodules of spindle cells arranged around blood vessels and capillaries of varying caliber (hematoxylin–eosin stain, original magnification X40). (**b**) The nodules are separated by vascular fibrocollagenous bundles (hematoxylin–eosin stain, original magnification X100)

**Fig. 3:**
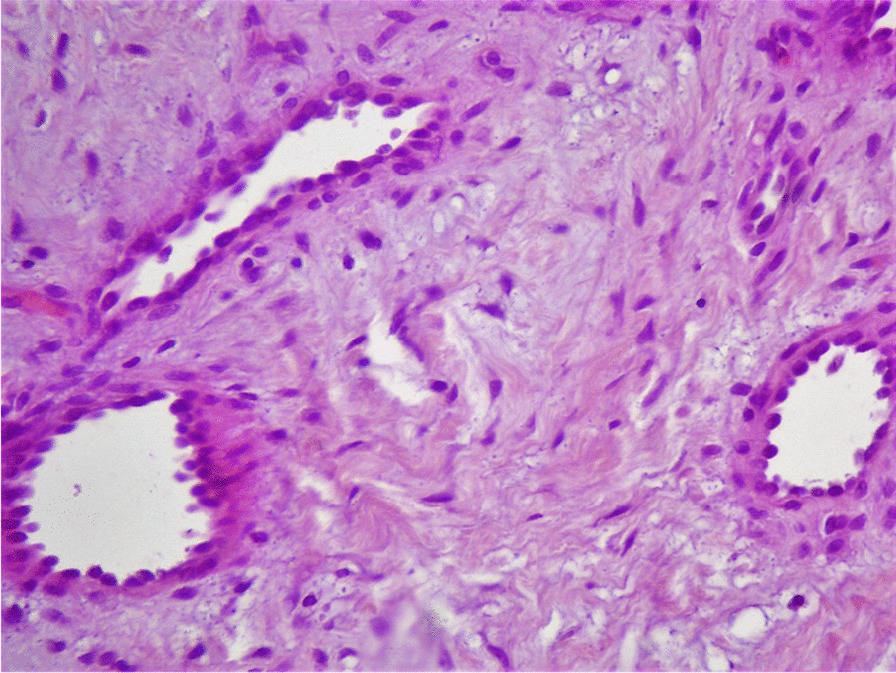
The nuclei of the lesion are spindled with eosinophilic cytoplasm. No necrosis or atypia was demonstrated (hematoxylin–eosin stain, original magnification X100)

**Fig. 4:**
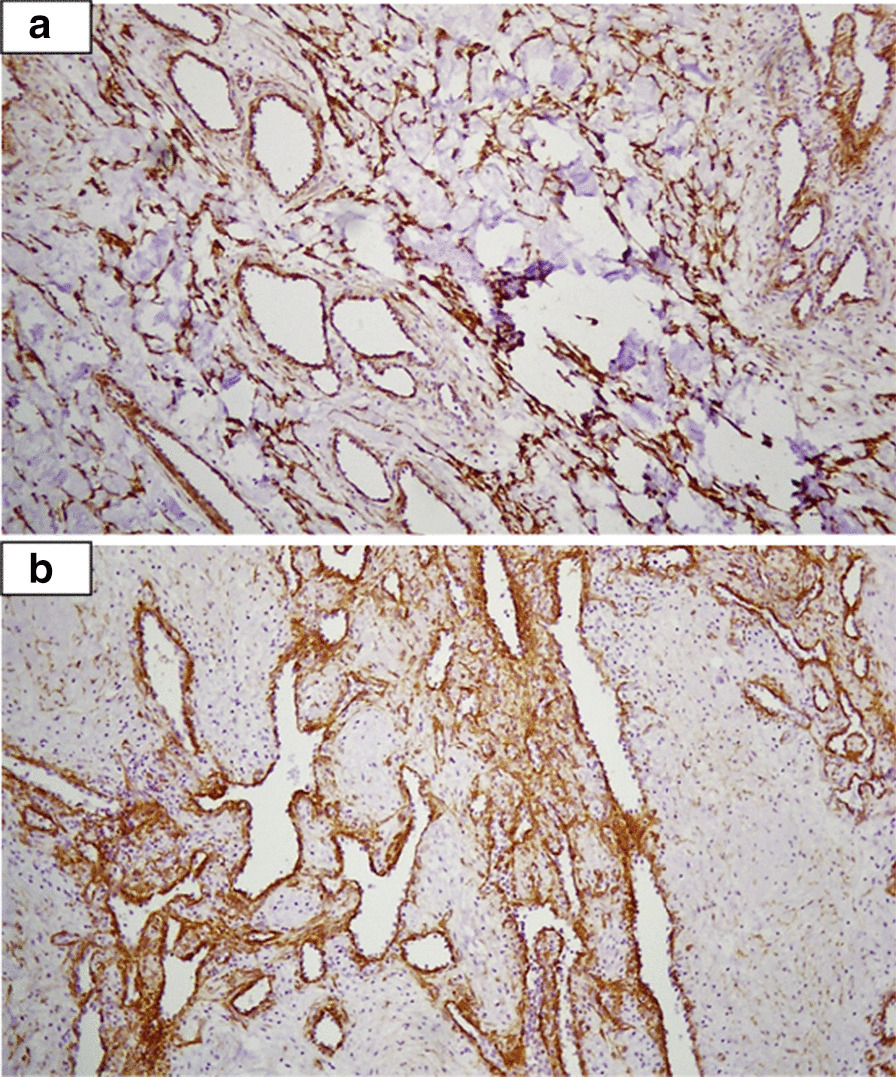
Calponin staining (A X100) and smooth muscle actin staining (BX100) of spindle cells and endothelial cells in Myopericytoma

Owing to the obscure nature of the lesion and the young age of the patient, an incisional biopsy was performed immediately under local anesthesia and sent urgently to the pathological department (one of the considered and alarming differential diagnosis being Lymphoma and Leukemia). After receiving the pathological report based on the incisional biopsy (diagnosis of MPC), the patient underwent excisional biopsy of the lesion under general anesthesia, and a diagnosis of MPC was once again established based on histological and immunohistochemical analysis (Figs. 2, 3, 4). The incisional and excisional biopsy were performed by the Principal Investigator (Prof. Abu El-Naaj, an Oral and Maxillofacial Surgeon). Types of suture that were used (incisional and excisional biopsy, respectively) were Silk 3*0 and Vicryl 4*0, single interrupted. The excisional biopsy procedure was performed under general anesthesia in order to relieve the patient’s anxiety and also in order to ensure the excision of the lesion and closure of the site are performed under the best medical conditions and maximal control. The operation itself lasted about an hour. Postoperatively, the patient received antibiotics (amoxicillin) and analgesics.

No further treatment was necessary as the lesion was fully excised.

**Fig. 5:**
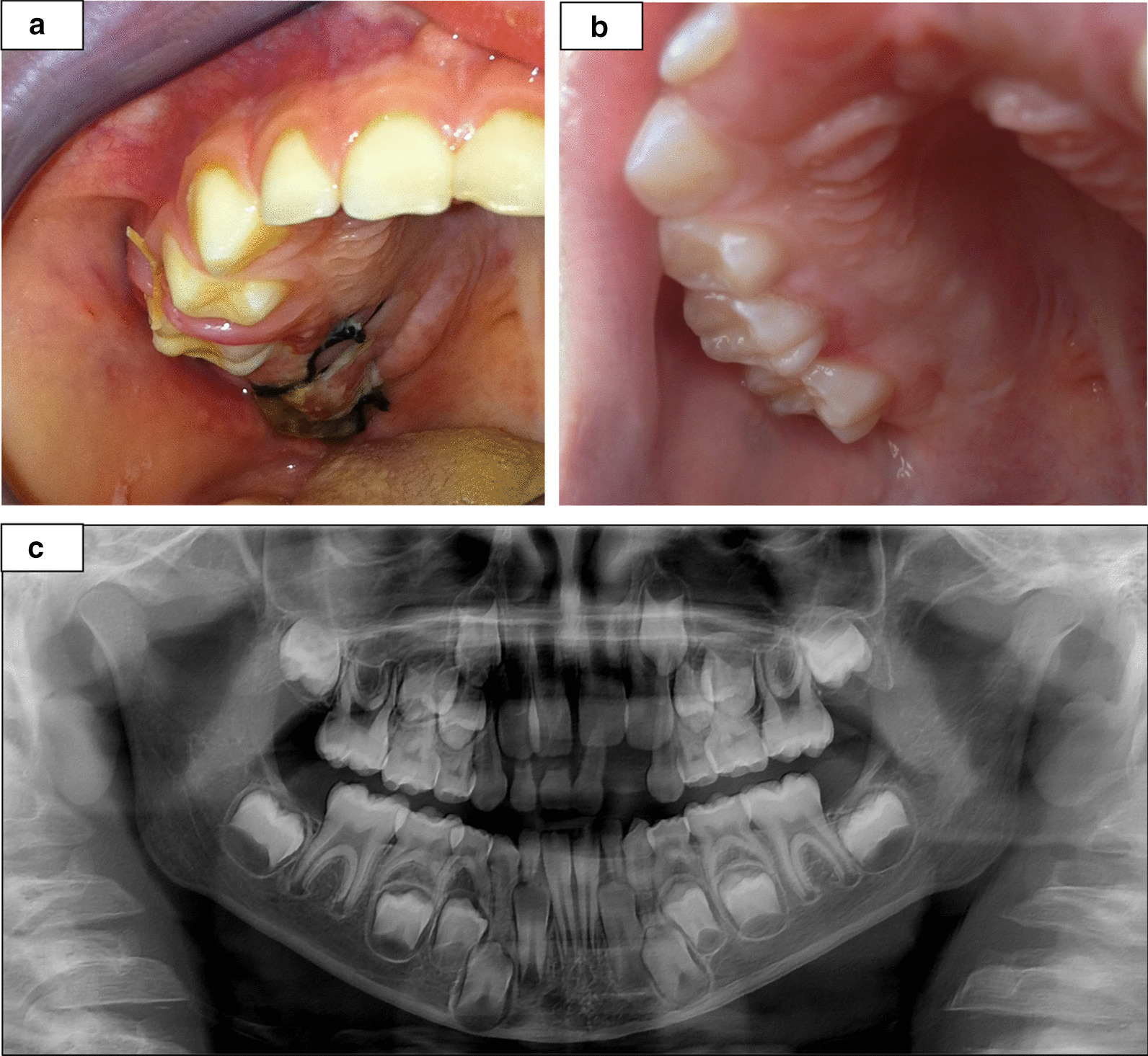
Healing and follow up: Uneventful healing of the surgical site. View of the site 2 days after the incisional biopsy (**a**). Clinical (**b**) and radiological (**c**) follow up 2 years postoperatively with no signs of recurrence

After the final diagnosis was established and the postoperative healing was uneventful (Fig. 5a), the patient remained under close follow up (Fig. 5b). During the first year the patient was examined every 3 months and later on, once a year. During each follow up session, clinical examination was performed. Once a year, a panoramic Xray was performed.

To present date, 8 years postoperatively, no recurrence was detected.

## Discussion and conclusions

MPC is a slow-growing benign neoplasm composed of cells that show apparent differentiation towards spindle-shaped perivascular myoid cells called myopericytes [1, 12].

The term myopericytes describes neoplastic pericytes exhibiting smooth muscle differentiation around vascular channels [13], thus- the neoplasm arising from these cells is consequently termed MPC [4, 14].

MPC of the oral cavity is a rare entity with only few cases reported in the literature: three were reported in the tongue [4, 15, 16], two in the buccal mucosa [1, 2] two in the lips [10, 17] and one in the alveolar mucosa [3] (Table 1).

**Table 1:** Summery of the studies that described oral myopericytoma

References	Age	Gender	Site	Treatment	Follow up	Immunohistochemistry stain	
Lau et al. [16]	42	Male	Tongue	Not specified	Not specified	+ −	α-smooth muscle actin Desmin
Akbulut et al. [15]	61	Female	Lateral tongue	Excision	No recurrence 18 month later	+ − − −	α-smooth muscle actin Desmin S-100 CD34
Datta et al. [4]	36	Female	Lateral tongue	Excision	Not specified	+	α-smooth muscle actin
Laga et al. [3]	72	Male	Alveolar mucosa	Excision	No recurrence 18 months later	+ + − −	α-smooth muscle actin h-caldesmon Desmin CD34
Ide et al. [1]	54	Female	Buccal mucosa	Excision	No recurrence 9 years later	+ + − −	α-smooth muscle actin h-caldesmon Desmin CD34
Terada et al. [2]	61	Male	Buccal mucosa	Excision	No recurrence 6 month later	+ − − −	α-smooth muscle actin Desmin S-100 protein CD34
Sapelli et al. [10]	28	Male	Upper lip	Excision	No recurrence 3 years later	+ −	α-smooth muscle actin Desmin
Vasenwala et al. [17]	14	Male	Upper lip	Excision	No recurrence	+ Weak	α-smooth muscle actin CD34

MPC shares histogenetic and morphologic traits with a spectrum of diseases such as glomus tumor, solitary fibrous tumors, myofibroma, and hemangiopericytoma [2, 4, 7, 12].

MPC was first described by Requena et al. in 1996 as an alternative designation for solitary myofibroma [14] and was adopted 2 years later by Granter, Badizadegan and Fletcher who described the MPC as a tumor of concentric periluminal proliferation of bland, round to ovoid cells [12]. In 2002 it was defined and classified by the World Health Organization as a soft tissue neoplasm with differentiation toward pericytic/perivascular tumors [6]. Later on, Granter et al. added the tumors with features of infantily-type myofibromatosis in adults, glomangiopericytoma (GPC), glomangiomyoma and angioleiomyoma (ALM) into the MPC category [12].

MPC can be found at any age between 10–87 years [5, 6, 18, 19] with a median age of 49 years [5, 6, 9, 18, 20, 21]. It is more common in males [9, 21] with a male to female ratio of 1.25:1 [20].

MPC is described as a subcutaneous nodule of 2 cm or less [11, 22]. MPC most commonly arises in the dermis or subcutaneous tissue of the extremities [1, 2, 4, 15, 23, 24]. The lower extremities are the most commonly affected site, followed by the upper extremities, the head and neck region and the trunk [10]. Oral presentation is extremely rare [1, 2, 4, 15].

It usually appears as a single well circumscribed, slow growing, painless nodule [9, 23, 24], although multiple lesions can occur [9, 21]. In addition, MPC can be multifocal involving multiple anatomic regions [3]. Multinodular tumors or deep-seated tumors behave more aggressively when compared to superficial nodules [17].

Malignant transformation was reported once in the oral cavity [2].

MPCs have been reported to arise in immunodeficient patients. The clinical presentation of MPC in HIV positive patient is unique- occurring outside somatic soft tissue, including bronchus, tongue, vocal cord, brain, hepatobiliary system and spinal epidural tissue [16, 25]. In addition, an association between EBV and immunosuppression was first reported by Calderaro in 2008 [26], however EBV-MPCs are uncommonly reported [25].

Being a rare entity, MPC can be easily misdiagnosed. Histological differential diagnosis include myofibroma, myofibromatosis, glomus tumor, perivascular epithelioid cell tumor, and hemangiopericytoma [27]. According to the literature, preoperative MRI and ultrasound investigations are found to be insufficient or misleading sometimes [28, 29].

Excisional biopsy and histological examination are mandatory for a definitive diagnosis. Perivascular myoid cells are seen arranged concentrically around thin-walled vascular channels [19], but these findings must be further confirmed by immunohistochemical analysis where myopericytes are immunoreactive for smooth muscle actin, CD34 and calponin but rarely for desmin [3, 30].

Application of strict morphologic criteria and appropriately selective immunohistochemical markers which were mentioned earlier will help in distinguishing MPC from its alike in the oral cavity [1, 4, 15, 17].

MPC is treated by surgical excision with good prognosis. Wide local excision is the recommended treatment to prevent recurrences, with a careful follow-up [31].

Recurrences are rare [24], most likely due to poor circumscription of the lesion, extension of the tumor beyond the main lesion or malignancy [19].

There is a considerable debate regarding the treatment of oral MPC. Either local excision or wide local excision may be the choice of treatment. One should take into consideration many factors, such as the age of the patient and the region of the lesion; buccal, lingual or labial mucosa may undergo wide local excision without any serious consequences. The described case of a 6 years old boy with a maxillary lesion presented a surgical challenge. A true wide local excision in this case mandates posterior maxillectomy, with significant mutilation and morbidity. Therefore, excision of the lesion was the definite treatment, with a very strict follow-up, including imaging of panoramic x-rays. In addition, fiber optic observation, in order to refute the suspicions that the tumor has penetrated/invaded into the maxillary sinus was performed.

In conclusion, conservative approach should be considered for the treatment oral MPC especially in young patients in tooth bearing areas. More studies are required in order to achieve established conclusions.

## Data Availability

Not applicable.

## Abbreviations

MPC: Myopericytoma
ASA: American Society of Anesthesiologists physical status
GPC: Lomangiopericytoma
ALM: Angioleiomyoma
CBCT: Cone beam computed tomography

## References

[1] Ide F, Obara K, Yamada H, Mishima K, Saito I (2007). Intravascular myopericytoma of the oral mucosa: a rare histologic variant in an uncommon location. Virchows Arch.

[2] Terada T (2012). Myopericytoma of low grade malignancy in the oral cavity. Rare Tumors.

[3] Laga AC, Tajirian AL, Islam MN (2008). Myopericytoma: report of two cases associated with trauma. J Cutan Pathol.

[4] Datta V, Rawal YB, Mincer HH, Anderson MK (2007). Myopericytoma of the oral cavity. Head Neck.

[5] Folpe AL, Lane KL, Paull G, Weiss SW (2000). Low-grade fibromyxoid sarcoma and hyalinizing spindle cell tumor with giant rosettes: a clinicopathologic study of 73 cases supporting their identity and assessing the impact of high-grade areas. Am J Surg Pathol.

[6] Fletcher CD, Unni KK, Mertens F. *Pathology and genetics of tumours of soft tissue and bone.* Vol 4: Iarc; 2002.

[7] Mentzel T, Dei Tos AP, Sapi Z, Kutzner H (2006). Myopericytoma of skin and soft tissues: clinicopathologic and immunohistochemical study of 54 cases. Am J Surg Pathol.

[8] Matsuyama A, Hisaoka M, Hashimoto H (2007). Angioleiomyoma: a clinicopathologic and immunohistochemical reappraisal with special reference to the correlation with myopericytoma. Hum Pathol.

[9] Aung PP, Goldberg LJ, Mahalingam M, Bhawan J (2015). Cutaneous myopericytoma: a report of 3 cases and review of the literature. Dermatopathology (Basel).

[10] Sapelli S, Ribas M, Martins WD, de Noronha L, Gomes AP (2009). Myopericytoma of the lip: report of case. Head Neck.

[11] McMenamin M, Fletcher C (2002). Malignant myopericytoma: expanding the spectrum of tumours with myopericytic differentiation. Histopathology.

[12] Granter SR, Badizadegan K, Fletcher CD (1998). Myofibromatosis in adults, glomangiopericytoma, and myopericytoma: a spectrum of tumors showing perivascular myoid differentiation. Am J Surg Pathol.

[13] Dictor M, Elner Å, Andersson T, Fernö M (1992). Myofibromatosis-like hemangiopericytoma metastasizing as differentiated vascular smooth-muscle and myosarcoma: myopericytes as a subset of myofibroblasts. Am J Surg Pathol.

[14] Requena L, Kutzner H, Hügel H, Rütten A, Furio V (1996). Cutaneous adult myofibroma: a vascular neoplasm. J Cutan Pathol.

[15] Akbulut S, Berk D, Demir MG, Kayahan S (2013). Myopericytoma of the tongue: a case report. Acta Medica (Hradec Kralove).

[16] Lau PP, Wong O-K, Lui PC (2009). Myopericytoma in patients with AIDS: a new class of Epstein-Barr virus-associated tumor. Am J Surg Pathol.

[17] Vasenwala SM, Afroz N, Ansari HA, Khan AH, Basari R, Rehman S (2015). Myopericytoma of lip: a rare lesion in an unusual location. Indian J Pathol Microbiol.

[18] Chu Z-G, Yu J-Q, Yang Z-G, Zhu Z-Y, Yuan H-M (2009). Myopericytoma involving the parotid gland as depicted on multidetector CT. Korean J Radiol.

[19] Mathew NK, Zhang KY, Batstone MD (2015). Myopericytoma of the coronoid process: a case report and review of the literature. Oral Maxillofac Surg Cases.

[20] Wu F, Sun J, Dong J, Wang X, Gao Q (2013). Management of multicentric myopericytoma in the maxillofacial region: a case report. Case Rep Oncol.

[21] Agrawal N, Nag K (2013). Myopericytoma of the thoracic spine: a case report and review of literature. Spine J.

[22] Zhang Z, Yu D, Shi H, Xie D (2014). Renal myopericytoma: a case report with a literature review. Oncol Lett.

[23] McMenamin ME, Calonje E (2002). Intravascular myopericytoma. J Cutan Pathol.

[24] Kara A, Keskinbora M, Kayaalp ME, Seker A, Erdil M, Bulbul M (2014). An atypical presentation of myopericytoma in palmar arch and review of the literature. Case Rep Orthop.

[25] Khaba M, Ramdial P, Pillay B, Steyn A, Nargan K (2016). Epstein-Bar r virus-associated myoid tumors in human immunodeficiency virus-infecte d patients. J AIDS Clin Res.

[26] Calderaro J, Polivka M, Gallien S (2008). Multifocal Epstein Barr virus (EBV)-associated myopericytoma in a patient with AIDS. Neuropathol Appl Neurobiol.

[27] Xia L, Chen Y, Geng N, Jiang J, Yang M, Zhang W (2010). Multifocal myopericytoma in the maxillofacial region: a case report. Oral Surg Oral Med Oral Pathol Oral Radiol Endod.

[28] Woollard A, Southgate C, Blair J (2007). Intravascular myopericytoma of the superficial palmar arch. J Hand Surg (European Volume).

[29] Uchida Y, Kuriyama M, Yoshida Y, Yano A, Orihashi K (2012). Diagnosis and surgical management of the arterial myopericytoma. J Plast Reconstr Aesthet Surg.

[30] Van Besien J, Uvin P, Van den Broecke C, Creytens D, Merckx L (2016). Renal metastasis of a malignant myopericytoma: a case report and review of literature. Eur Med J Urol.

[31] Bates AS, Craig P, Knepil GJ. Myopericytoma of the parotid region treated by extracapsular dissection. BMJ Case Rep. 2014;2014. 10.1136/bcr-2013-201924 PMC3987211 24717593

